# Desert Tortoises (*Gopherus agassizii*) Are Selective Herbivores that Track the Flowering Phenology of Their Preferred Food Plants

**DOI:** 10.1371/journal.pone.0116716

**Published:** 2015-01-30

**Authors:** W. Bryan Jennings, Kristin H. Berry

**Affiliations:** 1 Departamento de Vertebrados, Museu Nacional, Universidade Federal do Rio de Janeiro, Rio de Janeiro, RJ, 20940-040, Brazil; 2 U.S. Geological Survey, Western Ecological Research Center, Falcon Business Park, 21803 Cactus Avenue, Suite F, Riverside, California, 92518, United States of America; University of Stellenbosch, SOUTH AFRICA

## Abstract

Previous studies of desert tortoise foraging ecology in the western Mojave Desert suggest that these animals are selective herbivores, which alter their diet according to the temporal availability of preferred food plants. These studies, however, did not estimate availability of potential food plants by taking into account the spatial and temporal variability in ephemeral plant abundance that occurs within the spring season. In this study, we observed 18 free-ranging adult tortoises take 35,388 bites during the spring foraging season. We also estimated the relative abundance of potential food plants by stratifying our sampling across different phenological periods of the 3-month long spring season and by different habitats and microhabitats. This methodology allowed us to conduct statistical tests comparing tortoise diet against plant abundance. Our results show that tortoises choose food plants non-randomly throughout the foraging season, a finding that corroborates the hypothesis that desert tortoises rely on key plants during different phenological periods of spring. Moreover, tortoises only consumed plants in a succulent state until the last few weeks of spring, at which time most annuals and herbaceous perennials had dried and most tortoises had ceased foraging. Many species of food plants—including several frequently eaten species—were not detected in our plant surveys, yet tortoises located these rare plants in their home ranges. Over 50% of bites consumed were in the group of undetected species. Interestingly, tortoises focused heavily on several leguminous species, which could be nutritious foods owing to their presumably high nitrogen contents. We suggest that herbaceous perennials, which were rare on our study area but represented ~30% of tortoise diet, may be important in sustaining tortoise populations during droughts when native annuals are absent. These findings highlight the vulnerability of desert tortoises to climate change if such changes alter the availability of their preferred food plants.

## Introduction

Populations of Agassiz’s desert tortoise (*Gopherus agassizii*) have dramatically declined over the past several decades [1–3]. In response, the U.S. Fish and Wildlife Service listed the species as threatened, prepared a recovery plan, and designated critical habitat [1–4]. Tortoises are long-lived and spend over 90% of their lives underground in burrows or caves [5], escaping harsh desert conditions. They emerge to feed or drink when forage and water are available [6]. The seasonal pattern of activities above ground depends on location within the geographic range, because climate, habitat type, forage species and seasonal availability vary within the geographic range [2]. Some desert regions receive primarily winter rains, producing a flush of annual wildflowers in spring, whereas other regions receive both winter and summer rains, resulting in two seasons when succulent green annuals are available as forage. Droughts are common in all habitats where they live.

Among many serious threats to continued survival of the species are habitat destruction and alteration due to urbanization; agriculture; renewable energy and mineral development; highway and utility corridors; livestock grazing; and recreational vehicle use [1, 2]. These disturbances to the Mojave and western Sonoran desert ecosystems where the Agassiz’s desert tortoise lives promote the invasion and proliferation of introduced plant species [7–10] and increase the frequency of fires [7, 9, 11], both of which diminish tortoise habitat quality by negatively affecting the availability of forage plants and shrub cover essential for protection from the extremes of temperature and predators [12–15].

Researchers have speculated on the existence of a link between decreased forage quality and decreased tortoise nutrition and health [16, 17]. However, evidence that tortoises are selective foragers and rely on key forage plants is limited. In the western Mojave Desert, where rainfall occurs primarily in winter months, four studies of tortoise food habits suggest that adult and juvenile tortoises prefer certain winter annual and herbaceous perennial plants known as “ephemerals” [12, 13, 14, 18]. However, only two of these studies [13, 18] attempted to assess the availability of potential forage plants. Such estimates of food availabilty represent an important component in foraging studies because without this information it is not possible to ascertain whether or not an animal is selecting some food items over others. Although some studies [13, 18] provide compelling data to support the idea that desert tortoises are indeed selective herbivores, each study suffers from two important limitations. One shortcoming is that these studies encompassed a fraction of the three-month foraging season: one study lasted for a month [13] and the other for only seven days [18]. As a consequence, tortoise food habits and food plant availability at other times of the foraging season were unknown. Secondly, both studies only assessed plant availability along the foraging routes of tortoises, and thus did not explicitly consider, in a broader sense, the hierarchical nature of resource selection. If tortoises are selecting their foraging paths because their preferred foods are more abundant along these routes compared to surrounding areas, then resulting estimates of food plant “availability” as determined by the investigator may not be comparable to the actual scale of selection determined by the animal [19]. Hence, such comparisons of availability with diet choice could lead to spurious inferences about diet selection.

One major challenge to estimating the availability of potential forage plants is the tremendous spatial variation in annual plant diversity and abundance [13, 17]. At large scales, landscapes in the Mojave Desert are heterogeneous with alluvial fans or piedmonts, upland terrain with mountains and large hills, and stream channels [20]. On a smaller scale, the diversity and biomass of annual plants varies among different microhabitats. In years of above average rainfall, a luxuriant growth of annual plants occurs beneath the shaded or partially-shaded canopy of creosote bushes (*Larrea tridentata*) and other large shrubs in coppice mounds or islands of fertility [21]; the area encircling the outer edge of the canopy zone or “dripline” has noticeably lower biomass than the canopy zone; and the space between shrubs or “intershrub space” tends to have lower annual biomass [22].

Annual plant diversity and abundance can also vary considerably both among and within years depending on timing and amount of rainfall [17, 23, 24]. Few annuals germinate and flower during dry years, whereas spectacular displays of wildflowers appear during wet (i.e., El Niño) years. During wetter years, within-season variability in the diversity and biomass of annuals is due to the sequential process of emergence, flowering, and senescence of ephemeral plants that takes place over the several months-long growing season [23, 24]. Such changes in plant availability suggest that tortoises must consume different plant species during different time intervals or “phenological periods” of the spring growing season. In a study at a site in the western Mojave Desert, which spanned the spring foraging season during an El Niño year, adult tortoises apparently changed their food selections as spring progressed because ephemeral plant availability changed according to species-specific flowering phenologies [14]. Other tortoise species also have exhibited seasonal variations in diets [25–30]. Therefore, studies focused on tortoise diet should account for seasonal variation in diet as well as forage plant availability.

In this paper, we build on the foundation of previous work on desert tortoise foraging ecology by testing the null hypothesis that adult tortoises select forage plants in relation to their abundance throughout their entire foraging season. To accomplish this, we estimated the availability of ephemeral plants by taking into account both the spatial and temporal aspects of their availability in tortoise habitat. Our results strongly support the hypothesis that desert tortoises in the western Mojave Desert are selective herbivores. We also document other aspects of their foraging behavior and food preferences and discuss the conservation implications of our findings.

## Materials and Methods

### Study area

**Fig 1:**
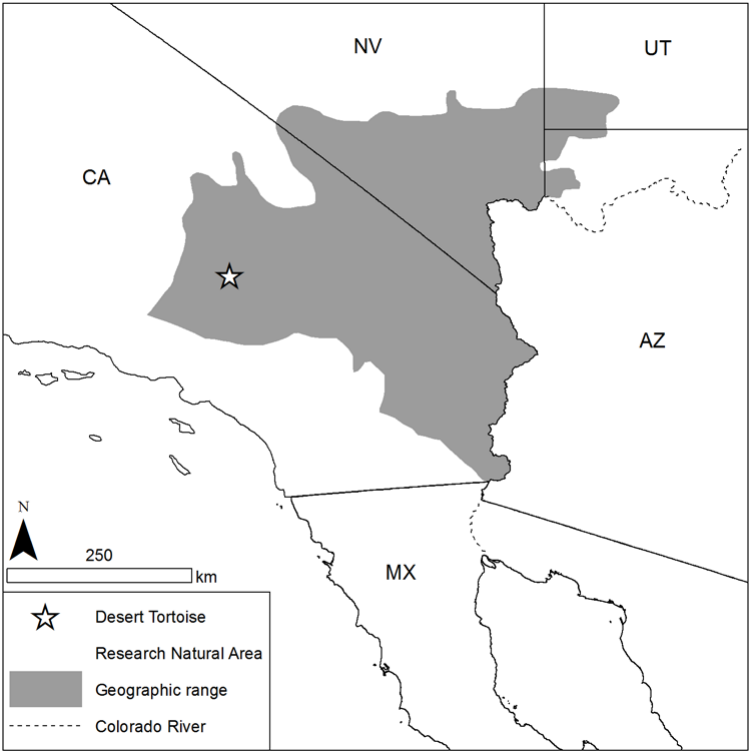
Location of the Desert Tortoise Research Natural Area (DTRNA) in eastern Kern County, California. The DTRNA is located at the western edges of the Mojave Desert and the geographical range of the desert tortoise.

This study was conducted at the Desert Tortoise Research Natural Area (DTRNA), a 100 km^2^ nature preserve located in eastern Kern County, California (Fig. 1), between 1 March and 30 June 1992. Permission to conduct this study in this preserve was granted by the Desert Tortoise Preserve Committee, Inc. and by the U.S. Bureau of Land Management (Desert District Office, Moreno Valley, California). The study area, located at 35°14′34”N and 117°51′45”W, is a 15 km^2^ area ranging in elevation from 800 to 915 m along a southwest-northeast gradient. Historical rainfall data collected at the nearby (22 km by air) Randsburg weather station, (elevation 1088 m), shows that >78% of annual precipitation occurs between October and March (National Oceanic and Atmospheric Administration, National Climate Data Center, Randsburg weather station, 1990–1992). The 20-year annual norm was 139.2 mm; in fall and winter of 1991–1992, preceding our spring study, precipitation totalled 276.9 mm, far exeeding the norm and producing a profusion of winter annual and herbaceous perennial species.

The DTRNA is home to at least 126 annual plant species, making this one of the most species-rich sites known in the Mojave Desert [24]. Nearly all of the annuals are “winter-spring” annuals, which flower from February to June. The species richness of perennial plants is also high with at least 57 species known from the DTRNA. This high level of diversity can be attributed to an ecotone between the Mojave and Great Basin deserts and the southern Sierra Nevada and Tehachapi Mountain ranges, as evident by the admixture of common Mojave Desert plants such as creosote bush, white bur-sage (*Ambrosia dumosa*), and the Joshua tree (*Yucca brevifolia*) with typically Great Basin species such as spiny hopsage (*Grayia spinosa*) and Anderson thornbush (*Lycium andersonii*). In contrast to the southern and eastern regions of the Mojave Desert, cacti and perennial bunchgrasses are less common.

### Estimation of food plant availability

To determine whether desert tortoises select particular plants for eating or instead consume plants in relation to their availability (i.e., non-selective foragers), we estimated the relative abundances of perennial and annual plants. However, to ensure that comparisons between estimates of food plant “availability” are comparable to estimates of food plant selections, it is essential to measure both variables on a common spatial scale [19]. Observations of adult tortoises conducted at this study area in 1991 by one of us (WBJ) suggested that tortoise home ranges and daily foraging routes usually encompassed all local habitat types (see below for definitions of habitat strata). Given that adult tortoise foraging routes appeared to often traverse all major habitat types found on the study area, we define availability of potential food plants as those plants occurring across the various habitat strata in which tortoises can be found.

The abundance of plants at the study area occurs on at least two scales of spatial heterogeneity. First, several topographical strata exist including a broad, sandy alluvial fan, low rocky and gravelly hills, and ephemeral stream channels of different sizes. These habitat strata influence the spatial distribution of most perennial and annual plant species [20]. Second, the distribution of annuals varies according to microhabitats within strata such as under a shrub canopy or within dripline zones and intershrub spaces [8, 22]. We therefore opted to sample the perennial and annual vegetation using a stratified random sampling methodology [31]; we stratified our samples in proportion to the relative area occupied by each stratum and by each microhabitat.

**Definition of habitat strata**. We defined three topographical and vegetational strata: (1) “hills,” areas of low rolling hills with a relatively undeveloped, rocky soils; (2) “alluvial fans,” transitional areas between the hills and stream channels composed of gravelly or sandy soils and occurring in low-lying areas; and (3) “stream channels,” sandy drainage areas 1–10 meters wide including edges and banks. We also noted the existence of a fourth stratum, which we refer to as “small stream channels,” hereafter considered as a sub-stratum of the stream channel stratum. These small sandy stream channels are typically 1–2 m wide and occur throughout the study area. These small stream channels may be relevant to tortoise foraging ecology, as one study [13] reported that adult tortoises often traveled along and foraged within these channels. Unfortunately, it was not possible for us to estimate the amount of area occupied by this habitat type due to the relatively small amount of area occupied by these small stream channels. Therefore, for our diet selection analyses we grouped small stream channels within the stream channel stratum. However, given the potential importance of this sub-stratum to desert tortoise foraging biology, we considered this sub-stratum in other analyses (see below).

**Fig 2:**
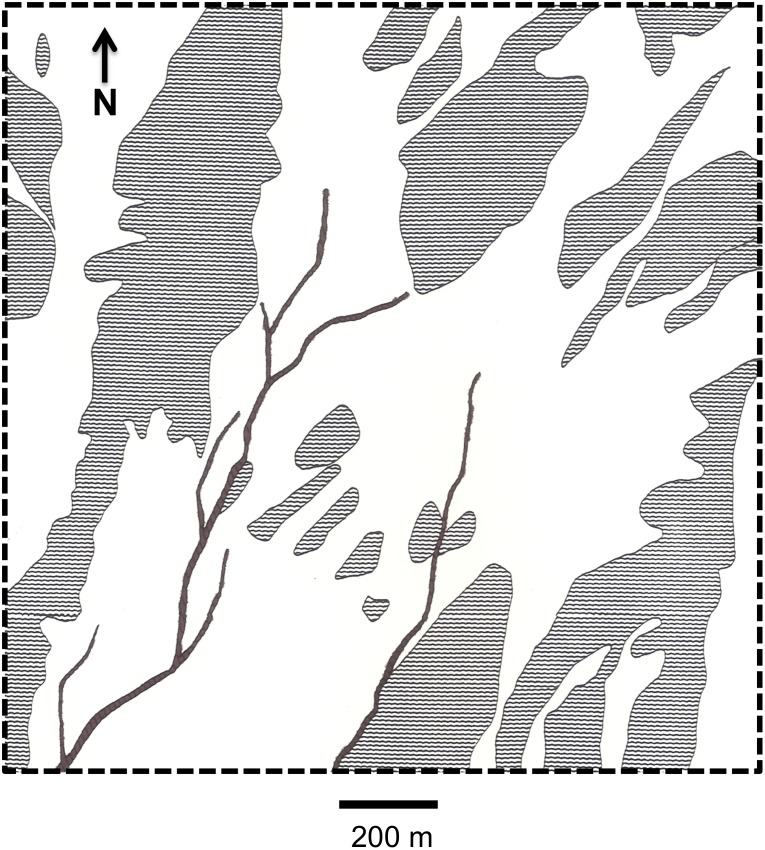
Topographical and vegetational map of the study area at the Desert Tortoise Research Natural Area. Defined habitat strata within this area include: “alluvial fans” (white), “hills” (gray stipple), and “stream channels” (black). Note, “small stream channels” (1–2 m wide), which occur sparsely throughout the study site, are not shown. The black dashed line delineates the study area where plant diversity and abundance was estimated.

We designated a 2.59 km^2^ area as our plant sampling area because it contained many of the study animals’ home ranges and because this area included representative patches of the previously defined habitat strata. Using aerial photos, we constructed a topographical and vegetational map of the area, which we later confirmed with field observations (Fig. 2). From this map, we calculated the percentage of area occupied by each stratum using a planimeter, which enabled us to proportionally allocate plant sampling quadrats among strata: alluvial fans = 48%, hills = 42%, and stream channels = 10%.

**Sampling of perennial plants**. A minimum of 1000 m^2^ should be sampled to accurately establish abundance (number of plants per 1000 m^2^) and cover (m^2^) of perennial species in Mojave Desert vegetation [32]. Although our primary objective was to obtain an estimate of the relative abundance (i.e., availability) of each perennial species occuring in the study area, some of our analyses also required estimates of the cover of each species (see below). We therefore designated our sample size as consisting of one hundred 10 m^2^ quadrats (1 quadrat = 2m x 5m), which in total covers 1000 m^2^. Quadrats were then proportionally allocated among habitat strata: alluvial fans = 48, hills = 42, and stream channels = 10. In each stratum, we randomly placed quadrats along a linear transect. Within each quadrat, we counted all species of perennial plants and measured the dimensions (i.e., major diameter, minor diameter, and height) of each individual plant. A plant was counted if its stem was contained within the quadrat; thus plants with their stems outside the quadrat but canopies partially within the quadrat were not counted. We computed plant cover for each plant by assuming that the coverage area was circular. We estimated cover (m^2^) for individual plants by taking the average of the two diameter estimates (maximum + minor dimeters)/2 and then input the resulting radius (m) value into the formula for the area of a circle. We then summed cover estimates for each species across quadrats in each stratum to generate an estimate of the absolute cover (m^2^) by species.

**Sampling of annual plants**. We chose a total of 26 sample quadrats because the high abundance of annuals made a larger sample size impractical. Each quadrat sample unit was 0.25 m^2^ (we note that even this size quadrat could contain >1000 plants). Owing to the topographical heterogeneity, quadrats were allocated to each stratum in proportion to the size of each stratum: alluvial fans = 12 quadrats, hills = 11 quadrats, and stream channels = 3 quadrats. Within each stratum we established a 100 m linear transect and sampled the annuals at random locations along this transect corresponding to the number of quadrats per stratum. To account for spatial variation in the distributions of annuals at the smallest scale, we further sub-divided the 0.25 m^2^ quadrat into four 25 × 25 cm sub-quadrats each of which represented one of the four microhabitats: 1) open or intershrub space, 2) outer edge or dripline of creosote bushes, 3) under the “canopy” of creosote bushes, and 4) outer edges of “other” or non-*Larrea* species of shrub [8, 22]. Along stream channel transects, at each quadrat location, we randomly placed all four sub-quadrats within the stream channel; if a creosote bush was not in the vicinity, then we collected two intershrub and two dripline samples from the nearest non-*Larrea* shrubs. After each sub-quadrat was selectively placed in a particular microhabitat, all individual plants were collected, identified, counted, and classified as either “succulent” or “dry.” We defined a plant as succulent if it contained water (e.g., plants are succulent from the time they emerge until just after they flower) or dry if it did not contain water (e.g., plants that had senesced). Nomenclature for plant species followed [33].

After sampling of annuals was completed, the raw numbers of plants taken from each sub-quadrat were adjusted to account for the relative amount of area occupied by each microhabitat at each quadrat sampling location. For example, the raw “intershrub” sub-quadrat represented 25% of the quadrat sampling effort yet intershrub spaces covered nearly 80% of area occupied by all microhabitats. Thus an uncorrected estimate would tend to underestimate the abundance of intershrub annuals, while overestimating the numbers of plants growing in other microhabitats in the vicinity. We derived estimates for the relative amount of area covered by each microhabitat from our estimates of perennial plant coverage (see Results).

In years prior to this study, we observed seasonal variation in the flowering phenologies among different species of annual plants at the DTRNA [24]. Between late winter to early spring when environmental conditions were cool and moist, flowering annuals tended to be characterized by a group of dominant species, including representatives of the Asteraceae (*Lasthenia californica*), Boraginaceae (*Amsinckia tessellata*, *Cryptantha angustifolia*, *C. nevadensis*, *Pectocarya* spp., *Phacelia tanacetifolia*, *Pholistoma membranaceum*), and Brassicaceae (*Caulanthus lasiophyllus*, *Tropidocarpum gracile*, *Lepidium flavum*). In mid-spring, as conditions became warmer and drier, the early spring dominants fruited and senesced while a new crop of annuals began to flower. These mid-spring dominants included representatives of the Asteraceae (*Chaenactis carphoclinia*, *C. fremontii*, *Eriophyllum pringlei*), Boraginaceae (*Cryptantha circumcissa*), Loasaceae (*Mentzelia eremophila*), and Polygonaceae (*Chorizanthe watsonii*, *Chorizanthe brevicornu*, *Eriogonum gracillimum*, *E. nidularium*) [24]. Towards the end of the spring, most early- and mid-spring species senesced with only a few still in flower. At this late stage of the wildflower season, the dominants included species from the Euphorbiaceae (*Croton setigerus*), Polemoniaceae (*Eriastrum eremicum*), and Polygonaceae (*Chorizanthe rigida*, *Eriogonum angulosum*, *E. gracillimum*, *E. nidularium*) [24]. Thus, to attain greater resolution and understanding of both food availability and tortoise diet over the course of the entire annual plant growing and tortoise foraging seasons, we divided the spring season into three successive phenological periods: early spring or what we will term the “first phenological period,” commenced 1 March (before tortoises emerged from winter hibernation) and lasted until 30 April; mid-spring or the “second phenological period,” from 1 May to 31 May; and late spring or the “third phenological period,” from 1 June to 30 June. The annual plant flora was sampled once within each of these three periods, but observations were made throughout each of these three periods.

### Estimation of desert tortoise diet

**Study animals**. Authorization to conduct this strictly observational study of free-ranging desert tortoises, which are protected as a Federally Threatened Species, was granted under federal and state permits issued to one of the authors (KHB). Eighteen free-ranging adult desert tortoises (10 males and 8 females) were observed foraging during the spring of 1992. Fourteen of these individuals were equipped with telemetry transmitters by other teams of researchers studying the physiology and health of desert tortoises [6, 34, 35]. Female and male tortoises ranged in size from 210 to 239 mm and 179 to 280 mm in carapace length at the midline, respectively. We tracked the tortoises using a telemetry receiver, initiating observations of foraging tortoises when they emerged from winter hibernation burrows in mid-March. We continued these observations throughout spring until early summer, at which time tortoise foraging activities largely ceased and tortoises retreated to deep burrows because of the lack of water and succulent green plants and to avoid high summer temperatures [6]. Tortoises were located early in the day before they emerged from their cover sites (burrow or shrub) for daily activity. Once a tortoise emerged, it was followed at distances of 4–8 m. These distances were effective at minimizing disturbance to the tortoise, yet close enough to clearly observe (sometimes using binoculars) feeding habits and behavior [13]. When a tortoise was observed foraging, the following data were simultaneously recorded on mini-recorders: species of plant, number of bites of plant material (or other items), parts of the plant eaten (e.g., flowers, seeds), condition of the plant (e.g., succulent or dry), and microhabitat where the plant grew (i.e., margin of stream channel, intershrub space or under canopy of shrub). When a tortoise bit at a plant but did not take in plant material, the bite was not counted.

Desert tortoises typically started feeding soon after emerging from night-time cover sites and continued feeding as they traveled “foraging routes” through their home ranges until they ceased foraging activities for the day and retreated to cover sites. If we measured the entire foot-path distance a given tortoise traveled between consecutive cover sites, then we classified this observation as a “complete foraging route.” If we did not record the entire distance, then we considered this an “incomplete foraging route” in our analyses. For each complete or incomplete foraging route we also noted the actual distances the tortoise traveled, in meters, through each stratum type. Moreover, because it was easy to recognize the small 1–2 m wide stream channels described earlier, we calculated the distances traveled through these small stream channels. These data on strata and sub-strata use were used to evaluate our assumption that tortoises forage in various strata. Generally only one tortoise was observed per day in an effort to document all foods eaten along each foraging route.

### Statistical analyses

To test the null hypothesis that tortoises consume their forage plants in relation to plant abundance (i.e., are not selective), we compared the relative abundance of plants vs. tortoise diet with Fisher’s Exact Test [36] using the R statistical package [37]. We performed separate tests for annuals and perennials and by phenological period resulting in six tests. Although tortoise food intake was primarily quantified in the field using “bites” taken from individual plants, for these tests we instead used individual plants as the units of analysis for both plant abundance and tortoise diet. Thus, regardless whether an individual tortoise took one bite or 10 bites from a single plant, we regarded the event as the tortoise sampling a single plant. An assumption of this test of independence holds that individual tortoises do not have food preferences that differ from other tortoises; in such a scenario, a non-significant statistical result might lead to an erroneous conclusion that adult desert tortoises do not have preferences when in reality they do (i.e., each individual has its own distinctive food preferences). We evaluated this assumption by examining inter-individual variation in diet to determine whether or not tortoises were consistently choosing the same forage plants. Prior to performing these tests, we constructed 2-dimensional tables each having two columns (abundance of plants in the environment or “availabilty” vs. abundance of those plants in tortoise diet) and with rows containing each plant species. Owing to the large number of rows in each table, we conducted Monte Carlo simulations in R based on 2000 replicates to generate *P*-values for each test. A *P*-value < 0.05 was taken as statistical evidence that tortoises chose their food plants independently of the abundance of those plants.

## Results

### Perennial plant abundance and cover

Our sampling of the perennial vegetation across the study area indicated that each topographical stratum had a characteristic perennial plant assemblage. For example, the hill stratum had the highest species richness with at least 11 perennial species, whereas alluvial fan and stream channel strata contained at least five and six perennial species, respectively (Tables 1–3). Although the latter two strata had a similar number of species, the species composition between them only partly overlapped: *Grayia spinosa* occurred in the alluvial fan stratum but not stream channel, whereas *Ambrosia salsola* and *Scutellaria mexicana* were detected in the stream channel but were apparently absent from the alluvial fan stratum. Although *G. spinosa* and *S. mexicana* seemed to be uncommon and therefore may have been overlooked in some strata due to insufficient sampling, this cannot be said for *A. salsola*. Individuals of *A. salsola* accounted for 25% of the perennial species in the stream channel stratum and 25% of coverage, yet this species was absent from the alluvial fan stratum. This finding is not surprising because *A. salsola* is a commonly observed species in Mojave Desert stream channel habitats (WBJ and KHB pers. observations). Though creosote bush is one of the hallmark plants species of the Mojave Desert, it was not the most abundant perennial species within hill, stream channel, or alluvial fan strata (6%, 7%, 9%, respectively). However this species was the dominant species in terms of relative cover in all strata (47%, 46%, 67%, respectively). When samples from all strata were considered together, the most abundant perennial species on the study area were: *Ambrosia dumosa* (37%), *Acamptopappus sphaerocephalus* (32%), *Eriogonum fasiculatum* (8%), and *L. tridentata* (7%); all other species ranged from <1 to 4% in relative abundance (Table 4). S1 Table provides all data used to estimate abundances and cover values of perennial species as well the correction factors used to adjust annual plant counts among microhabitats.

**Table 1:** Estimates of perennial plant abundance, relative abundance, cover, and relative cover in the hill stratum sampled with 42 10 m^2^ quadrats at the Desert Tortoise Research Natural Area, eastern Kern County, California.

**Species**	**Abundance per 420 m^2^**	**Relative abundance (%)**	**Cover (m^2^)**	**Relative cover (%)**
*Ambrosia dumosa*	98	44.1	23.5	24.7
*Eriogonum fasiculatum*	34	15.3	3.5	3.7
*Acamptopappus sphaerocephalus*	30	13.5	4.9	5.1
*Xylorhiza tortifolia*	18	8.1	1.4	1.5
*Larrea tridentata*	13	5.9	44.7	47.1
*Eriogonum inflatum*	8	3.6	1.3	1.4
*Ambrosia salsola*	7	3.2	6.5	6.8
*Psorothamnus arborescens*	6	2.7	4.9	5.2
*Mirabilis laevis*	4	1.8	0.4	0.4
*Tetradymia stenolepis*	3	1.4	3.8	4.0
*Stephanomeria pauciflora*	1	0.5	0.2	0.2
Total	222	100.0	94.9	100.0

**Table 2:** Estimates of perennial plant abundance, relative abundance, cover, and relative cover in the alluvial fan stratum sampled with 48 10 m^2^ quadrats at the Desert Tortoise Research Natural Area, eastern Kern County, California.

**Species**	**Abundance per 480 m^2^**	**Relative abundance (%)**	**Cover (m^2^)**	**Relative cover (%)**
*Acamptopappus sphaerocephalus*	99	62.7	22.1	21.3
*Ambrosia dumosa*	41	26.0	9.2	8.9
*Larrea tridentata*	14	8.9	69.1	66.7
*Lycium andersonii*	3	1.9	2.0	1.9
*Grayia spinosa*	1	0.6	1.2	1.2
Total	158	100.0	103.5	100.0

**Table 3:** Estimates of perennial plant abundance, relative abundance, cover, and relative cover in the stream channel stratum sampled with 10 10 m^2^ quadrats at the Desert Tortoise Research Natural Area, eastern Kern County, California.

**Species**	**Abundance per 100 m^2^**	**Relative abundance (%)**	**Cover (m^2^)**	**Relative cover**
*Ambrosia dumosa*	17	37.8	5.6	16.0
*Ambrosia salsola*	11	24.4	8.7	25.0
*Acamptopappus sphaerocephalus*	8	17.8	2.0	5.9
*Scutellaria mexicana*	4	8.9	1.5	4.3
*Larrea tridentata*	3	6.7	16.1	46.2
*Lycium andersonii*	2	4.4	0.9	2.6
Total	45	100.0	34.8	100.0

**Table 4:** Estimates of perennial plant abundance, relative abundance, cover, and relative cover in the hill, alluvial fan, and stream channel strata combined sampled with 100 10 m^2^ quadrats at the Desert Tortoise Research Natural Area, eastern Kern County, California.

**Species**	**Abundance per 1000 m^2^**	**Relative abundance (%)**	**Cover (m^2^)**	**Relative cover (%)**
*Ambrosia dumosa*	156	36.7	38.2	16.4
*Acamptopappus sphaerocephalus*	137	32.2	29.0	12.4
*Eriogonum fasiculatum*	34	8.0	3.5	1.5
*Larrea tridentata*	30	7.1	129.8	55.7
*Ambrosia salsola*	18	4.2	15.2	6.5
*Xylorhiza tortifolia*	18	4.2	1.4	0.6
*Eriogonum inflatum*	8	1.9	1.3	0.6
*Psorothamnus arborescens*	6	1.4	4.9	2.1
*Lycium andersonii*	5	1.2	2.9	1.2
*Scutellaria mexicana*	4	0.9	1.5	0.6
*Mirabilis laevis*	4	0.9	0.4	0.2
*Tetradymia stenolepis*	3	0.7	3.8	1.6
*Grayia spinosa*	1	0.2	1.2	0.5
*Stephanomeria pauciflora*	1	0.2	0.2	0.1
Total	425	100.0	233.3	100

### First Phenological Period (1 March–30 April)

At least 42 species of annuals were flowering during the early spring period with a density of 1,502 plants per m^2^ (Table 5). The more abundant species were *Lasthenia californica*, *Schismus barbatus*, *Pectocarya spp*., and *Erodium cicutarium*, a group that comprised 75% of the plants counted (Table 5). Although these four species were among the more abundant annuals in each stratum, annual plant assemblages appeared to differ among strata in other ways. Species richness in hill stratum was most diverse (35 species), whereas the alluvial fan and stream channel strata had lower numbers of species (30 and 14 species, respectively, S2 Table). Compositional differences were also observed among strata; each strata had species not found in the other strata: six in the alluvial fan stratum, seven in the hill stratum, and one in the stream channel stratum (S2 Table). Densities of annuals also varied among strata: alluvial fan (2,918 plants/m^2^), hill (1,308 plants/m^2^), and stream channel (280 plants/m^2^; S2 Table).

**Table 5:** Estimates of abundance and relative abundance of annual plants during the first phenological period (1 March–30 April) at the Desert Tortoise Research Natural Area, eastern Kern County, California.

**Species**	**Abundance per m^2^**	**Relative abundance (%)**
*Lasthenia californica*	380.24	25.32
*Schismus barbatus*	304.72	20.29
*Pectocarya spp.*	282.85	18.83
*Erodium cicutarium*	160.93	10.72
*Cryptantha circumcissa*	70.29	4.68
*Chorizanthe watsoni*	49.58	3.30
*Chorizanthe brevicornu*	36.88	2.46
*Stylocline psilocarphoides*	34.52	2.30
*Amsinckia tessellata*	26.96	1.80
*Gilia minor*	19.08	1.27
*Eriophyllum pringlei*	17.58	1.17
*Caulanthus lasiophyllus*	15.93	1.06
*Nemacladus spp.*	14.39	0.96
*Eremothera boothii*	12.54	0.84
*Oxytheca perfoliata*	10.22	0.68
*Chaenactis carphoclinia*	8.89	0.59
*Loeseliastrum schottii*	7.65	0.51
*Eriogonum gracillimum*	6.69	0.45
*Centrostegia thurberi*	5.66	0.38
*Eriogonum nidularium*	4.76	0.32
*Chaenactis fremonti*	4.33	0.29
*Linanthus parryae*	3.96	0.26
*Eriastrum eremicum*	3.45	0.23
*Tetrapteron palmeri*	3.14	0.21
*Plantago ovata*	3.06	0.20
*Mentzelia spp.*	2.69	0.18
*Phacelia tanacetifolia*	2.03	0.13
*Cryptantha nevadensis*	1.83	0.12
*Prenanthella exigua*	1.34	0.09
*Eriogonum pusillum*	1.33	0.09
*Chorizanthe rigida*	1.13	0.07
*Linanthus dichotomus*	0.85	0.06
*Malacothrix coulteri*	0.60	0.04
*Phacelia fremonti*	0.55	0.04
*Syntrichopappus fremonti*	0.46	0.03
*Astragalus didymocarpus*	0.33	0.02
*Pholistoma membranaceum*	0.15	0.01
*Salvia columbariae*	0.12	0.01
*Bromus madritensis*	0.05	0.00
*Eremelche exilis*	0.02	0.00^‡^
*Malacothrix glabrata*	0.02	0.00^‡^
*Caulanthus inflatus*	0.01	0.00^‡^
Total	1,501.75	100.00

^‡^denotes trace abundance.

Adult tortoises emerged from winter hibernation burrows on 20 March and were observed to commence foraging activities on 24 March. During this early spring period, we recorded foraging observations from 10 adult tortoises. For nine of the tortoises (four females and five males), we obtained 22 complete foraging routes throughout this time interval (S3 Table). Data on distances traveled through habitat strata were obtained for 19 foraging routes, which averaged 267 m in actual length. In ten of these routes, tortoises remained in only one stratum, six routes included segments in two strata, and three routes had segments in all three strata. The relative amounts of travel within each stratum were: 16.2% in alluvial fans, 67.6% in hills, and 16.2% in stream channels (of which 12.2% occurred in small stream channels and 4.0% in larger stream channels). Tortoises were observed to eat plants in all strata.

Although some inter-individual variation in diet existed, seven of the nine tortoises for which complete daily foraging information were obtained fed most heavily on two species of herbaceous perennials: *Mirabilis laevis* and *Astragalus layneae*. Of the two tortoises that didn’t consume these two plant species, one only ate the annual *Phacelia tanacetifolia* while the other tortoise primarily fed on two leguminous annual species (*Astragalus didymocarpus* and *Acmispon brachycarpus*). The tortoise with incomplete foraging route data was only observed to eat *A. brachycarpus* (S3 Table). These data show that tortoises foraged upon few plant species from among the many that were available to them.

The tortoises consumed a total of 12,236 bites from 689 individual plants representing at least 36 plant species (32 annuals and 4 perennials; Table 6). All forage plants were in a succulent state. Annuals and perennials were similarly represented in their diet if measured using bites (54% annuals vs. 46% perennials), though a far larger number of individual annual plants were eaten (86%) compared to perennials (14%; Table 6). Overall, *M. laevis* and *A. layneae* represented nearly half of tortoise diets (45% of bites). Moreover, tortoises were never observed to walk past these two species without feeding on them as well as other favored food plants (e.g., *Astragalus didymocarpus* and *Eremothera boothii*).

**Table 6:** Foods eaten by desert tortoises during the first phenological period (1 March–30 April) at the Desert Tortoise Research Natural Area, eastern Kern County, California.

**Species**	**# Bites**	**# Plants**	**% Bites**	**% Plants**
***Mirabilis laevis***	3,638	31	29.73	4.50
***Astragalus layneae***	1,704	35	13.93	5.08
*Astragalus didymocarpus*	1,509	29	12.33	4.21
*Eremothera boothii*	708	96	5.79	13.93
*Phacelia tanacetifolia*	682	34	5.57	4.93
*Mentzelia spp.*	558	25	4.56	3.63
*Amsinckia tessellata*	373	29	3.05	4.21
*Gilia minor*	342	52	2.80	7.55
*Erodium cicutarium*	281	40	2.30	5.81
*Tetrapteron palmeri*	266	26	2.17	3.77
*Stylocline psilocarphoides*	235	56	1.92	8.13
*Malacothrix coulteri*	217	18	1.77	2.61
*Lupinus odoratus*	211	3	1.72	0.44
*Acmispon brachycarpus*	200	9	1.63	1.31
***Stephanomeria parryi***	131	18	1.07	2.61
*Malacothrix glabrata*	125	13	1.02	1.89
*Chaenactis fremontii*	119	21	0.97	3.05
*Cryptantha circumcissa*	107	26	0.87	3.77
*Loeseliastrum schottii*	79	8	0.65	1.16
*Plantago ovata*	78	10	0.64	1.45
*Pectocarya spp.*	75	11	0.61	1.60
*Tropidocarpum gracile*	75	2	0.61	0.29
*Linanthus dichotomus*	66	8	0.54	1.16
*Schismus barbatus*	50	7	0.41	1.02
*Allium fimbriatum*	25	5	0.20	0.73
*Unknown grass sp.*	18	1	0.15	0.15
*Pholistoma membranaceum*	14	1	0.11	0.15
*Chorizanthe rigida*	12	4	0.10	0.58
*Eriogonum gracillimum*	12	1	0.10	0.15
*Eriogonum pusillum*	12	1	0.10	0.15
*Bromus madritensis*	9	2	0.07	0.29
*Prenanthella exigua*	8	1	0.07	0.15
*Calycoseris parryi*	7	2	0.06	0.29
*Chorizanthe brevicornu*	2	1	0.02	0.15
***Lomatium mohavense***	2	1	0.02	0.15
*Chaenactis carphoclinia*	1	1	0.01	0.15
Unknown plant spp.	285	61	2.33	8.85
Total	12,236	689	100.00	100.00

Plant names in boldface are herbaceous perennials; # Plants refers to the total number of individual plants for a given species that tortoises fed from (i.e., a plant was counted if a tortoise made at least one bite from that plant).

**Fig 3:**
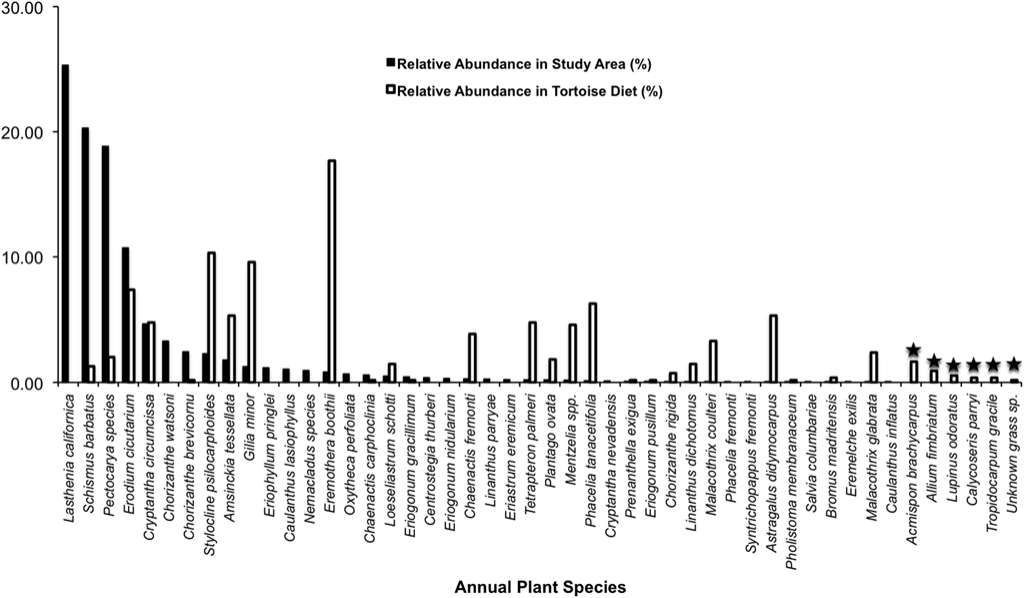
Tortoise diet in relation to annual plant abundance during early spring. Solid black bars show the relative abundance of each species found in the study area, whereas white bars indicate the relative abundance of each species in tortoise diet during the same period. Star symbols above bars denote plants in tortoise diet that were not detected in the plant survey.

**Fig 4:**
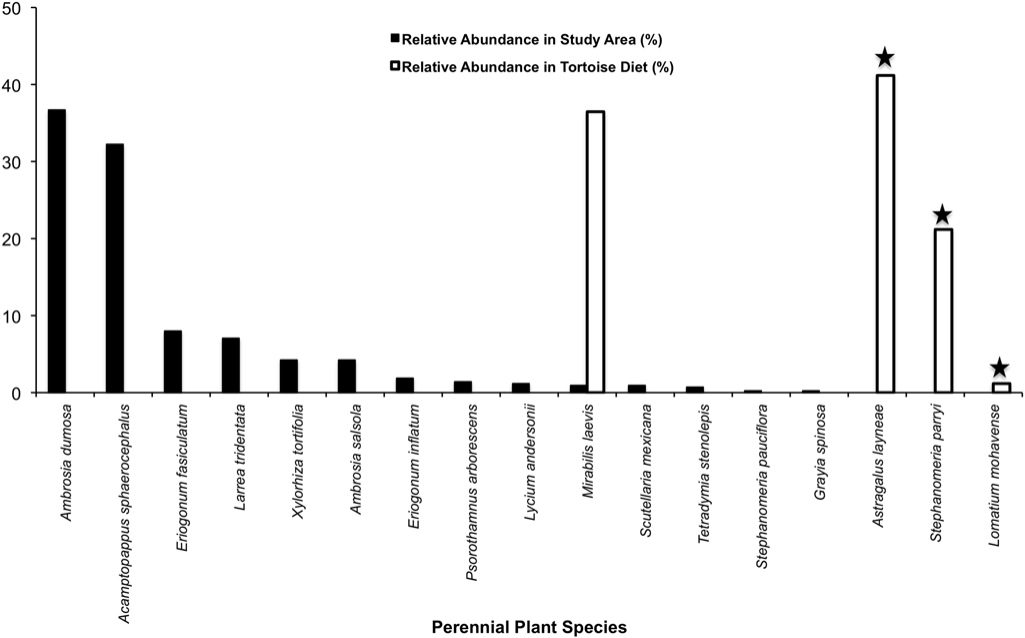
Tortoise diet in relation to perennial plant abundance during early spring. Solid black bars show the relative abundance of each species found in the study area, whereas white bars indicate the relative abundance of each species in tortoise diet during the same period. Star symbols above bars denote plants in tortoise diet that were not detected in the plant survey.

A comparison of relative abundance of annuals and perennials with their representation in tortoise diets shows that tortoises did not eat plants in relation to their abundance during the first phenological period (*P* = 0.0005; Figs. 3–4; S4–S5 Tables). Although 42 species of annuals and 14 species of perennials were counted in our stratified random samples, it is noteworthy that six species of annuals (*A. brachycarpus*, *Allium fimbriatum*, *Lupinus odoratus*, *Calycoseris parryi*, *Tropidocarpum gracile*, and an unidentified grass species) and three species of perennials (*A. layneae*, *Stephanomeria parryi*, and *Lomatium mohavense*) consumed by tortoises were not found in the plant surveys (Figs. 3–4; S4–S5 Tables).

### Second Phenological Period (1 May–31 May)

Results of annual plant sampling for this mid-spring period revealed 30 species of flowering annuals with the most abundant being *Chorizanthe watsoni*, *C. brevicornu*, *Erodium cicutarium*, *and Loeseliastrum schottii* and an overall density of 249 plants per m^2^ (Table 7). These estimates suggest that both the species richness and densities of annuals declined compared to early spring. However, similar to the first phenological period, the hill stratum contained the highest species richness with at least 24 species flowering there, followed by the alluvial fan and stream channel strata with 15 and 5 species, respectively (S6 Table). Among-strata differences in species composition were again observed to vary: the alluvial fan stratum contained five species not sampled in the other two strata—12 in the hill stratum and one in the wash stratum (S6 Table). Densities of annuals also varied among strata: alluvial fan (315 plants/m^2^), hill (398 plants/m^2^), and stream channel (35 plants/m^2^; S6 Table).

**Table 7:** Estimates of abundance and relative abundance of annual plants during the second phenological period (1 May–31 May) at the Desert Tortoise Research Natural Area, eastern Kern County, California.

**Species**	**Abundance per m^2^**	**Relative abundance (%)**
*Chorizanthe watsoni*	63.51	25.47
*Chorizanthe brevicornu*	43.88	17.60
*Erodium cicutarium*	27.92	11.20
*Loeseliastrum schottii*	22.12	8.87
*Cryptantha circumcissa*	16.05	6.44
*Eriophyllum pringlei*	12.78	5.13
*Lasthenia californica*	11.16	4.47
*Eriogonum gracillimum*	9.83	3.94
*Eriastrum eremicum*	9.72	3.90
*Chaenactis carphoclinia*	5.63	2.26
*Eremothera boothii*	4.05	1.62
*Nemacladus spp.*	3.89	1.56
*Syntrichopappus fremonti*	3.29	1.32
*Eriogonum nidularium*	3.01	1.21
*Chorizanthe rigida*	1.88	0.75
*Eriogonum pusillum*	1.78	0.71
*Oxytheca perfoliata*	1.68	0.67
*Centrostegia thurberi*	1.49	0.60
*Schismus barbatus*	1.13	0.45
*Chaenactis fremonti*	0.98	0.39
*Pectocarya spp.*	0.85	0.34
*Linanthus parryae*	0.70	0.28
*Astragalus didymocarpus*	0.49	0.20
*Gilia sp.*	0.38	0.15
*Prenanthella exigua*	0.38	0.15
*Malacothrix coulteri*	0.38	0.15
*Phacelia tanacetifolia*	0.29	0.12
*Stylocline psilocarphoides*	0.06	0.03
*Plantago ovata*	0.06	0.02
*Lupinus odoratus*	0.03	0.01
	249.37	100.00

During the second phenological period, we recorded foraging observations from ten adult tortoises. For seven tortoises (five females and two males), we obtained 17 complete foraging routes (S7 Table). Data on distances traveled through habitat strata were obtained for 12 routes and averaged 185 m in length. In 11 of these routes, tortoises remained in only one stratum and one route included two strata. The relative distances traveled through each stratum type were: 0.0% alluvial fans, 87.6% hills, and 12.4% stream channels (of which 5.6% was in small stream channels and 6.8% in larger stream channels).

In contrast to the first phenological period, variation among individuals in terms of the more frequently eaten plant species was less: *A. brachycarpus* was the most frequently eaten plant for six of the seven tortoises with complete foraging routes, while *Prenanthella exigua* was the most favored plant for the seventh tortoise (S7 Table). Of the three tortoises for which we only had partial daily foraging data, one mostly ate *M. laevis*, another primarily consumed *A. brachycarpus*, and the third was observed to only ingest the annual *Amsinckia tessellata* (S7 Table).

The ten tortoises were observed taking a total of 16,143 bites from 1,422 individual plants representing 17 species (14 annuals and 3 perennials; Table 8). Like the first phenological period, all food plants were in a succulent state. However, in contrast to the first phenological period, annual plants, especially *A. brachycarpus* and *P. exigua*, accounted for the vast majority of tortoise bites (91%; Table 8). Tortoises consumed fewer bites of *A. layneae* (7%) and *M. laevis* (<1%) than they did during the first phenological period (compare Tables 6, 8). Upon encountering individuals of any of the three most-eaten food plants (*A. brachycarpus*, *P. exigua*, and *A. layneae*), tortoises always stopped to consume all or part of the plants, similar to their behavior during the first phenological period when they fed on the four most frequently eaten plants (*M. laevis*, *A. layneae*, *A. didymocarpus*, and *Eremothera boothii*) whenever encountered.

**Table 8:** Foods eaten by desert tortoises during the second phenological period (1 May–31 May) at the Desert Tortoise Research Natural Area, eastern Kern County, California.

**Species**	**# Bites**	**# Plants**	**% Bites**	**% Plants**
*Acmispon brachycarpus*	10,312	999	63.88	70.25
*Prenanthella exigua*	1,969	133	12.20	9.35
***Astragalus layneae***	1,191	36	7.38	2.53
*Chorizanthe brevicornu*	541	32	3.35	2.25
*Cryptantha circumcissa*	420	30	2.60	2.11
*Erodium cicutarium*	391	63	2.42	4.43
*Plantago ovata*	352	56	2.18	3.94
*Eremothera boothii*	287	27	1.78	1.90
***Chamaesyce albomarginata***	200	4	1.24	0.28
*Amsinckia tessellata*	194	16	1.20	1.13
*Astragalus didymocarpus*	114	5	0.71	0.35
***Mirabilis laevis***	99	7	0.61	0.49
*Mentzelia spp.*	18	1	0.11	0.07
*Pectocarya spp.*	13	3	0.08	0.21
*Caulanthus inflatus*	8	1	0.05	0.07
*Astragalus acutirostris*	5	1	0.03	0.07
*Eriastrum eremicum*	5	1	0.03	0.07
Unknown plant spp.	24	7	0.15	0.49
Total	16,143	1,422	100.00	100.00

Plant names in boldface are herbaceous perennials; # Plants refers to the total number of individual plants for a given species that tortoises fed from (i.e., a plant was counted if a tortoise made at least one bite from that plant).

**Fig 5:**
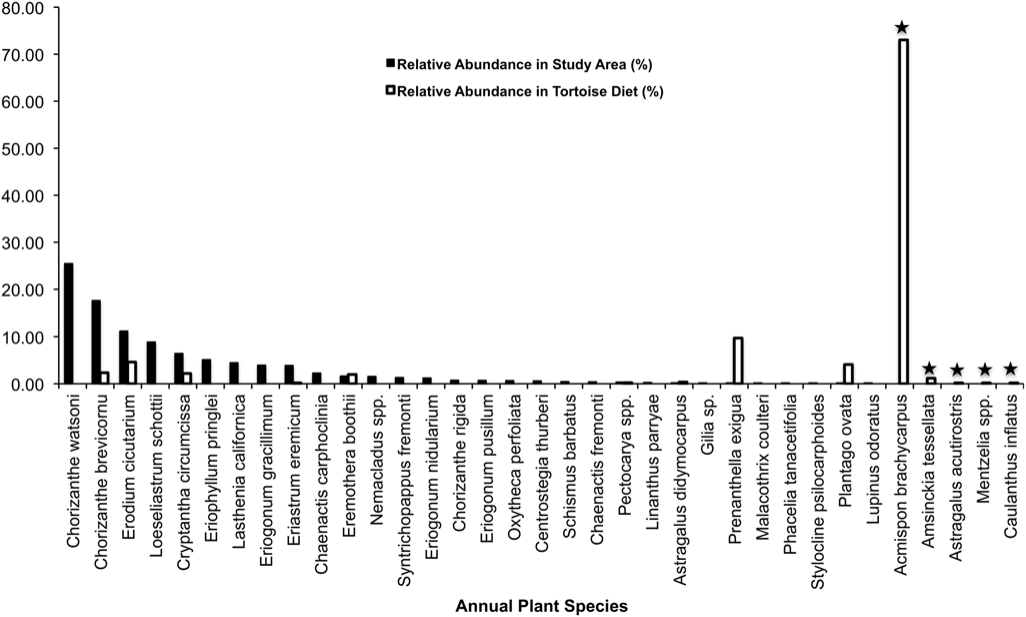
Tortoise diet in relation to annual plant abundance during mid-spring. Solid black bars show the relative abundance of each species found in the study area, whereas white bars indicate the relative abundance of each species in tortoise diet during the same period. Star symbols above bars denote plants in tortoise diet that were not detected in the plant survey.

**Fig 6:**
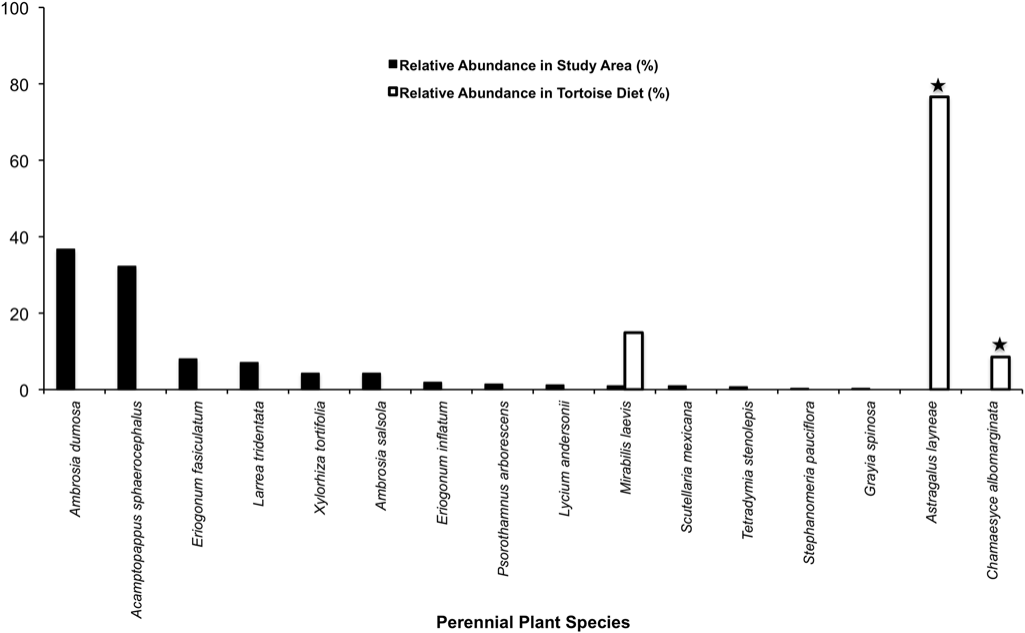
Tortoise diet in relation to perennial plant abundance during mid-spring. Solid black bars show the relative abundance of each species found in the study area, whereas white bars indicate the relative abundance of each species in tortoise diet during the same period. Star symbols above bars denote plants in tortoise diet that were not detected in the plant survey.

A comparison of relative abundance of annuals with their representation in tortoise diet shows, again, that tortoises did not eat annual and perennial plants in relation to their abundance during the second phenological period (*P* = 0.0005; Figs. 5–6; S8–S9 Tables). Despite our plant sampling efforts, which documented 30 annual and 14 perennial species, five species of annuals (including *Acmispon brachycarpus*, *Amsinckia tessellata*, *Astragalus acutirostris*, *Caulanthus inflatus*, and *Mentzelia spp*.) and two species of perennials (i.e., *A. layneae* and *Chamaesyce albomarginata*) consumed by tortoises were not detected in the plant surveys (Figs. 5–6; S8–S9 Tables).

### Third Phenological Period (1 June– 30 June)

The numbers of succulent annual plants available as forage for tortoises sharply declined by late spring: the estimated number of species in a succulent state was seven and the density of annual plants was nine per m^2^ (Table 9). Over 98% of the annual plants, represented by 39 species, were in a dried (senescent) state. In the hill stratum, only four annuals in a succulent state were encountered: *Eriastrum eremicum*, *Eriogonum angulosum*, *Eriogonum gracillimum*, and *Erodium cicutarium* (S10 Table). The alluvial fan stratum also contained at least four annuals in a succulent state and all were species of *Eriogonum*: *E. angulosum*, *E. gracillimum*, *E. nidularium*, and *E. pusillum* (S10 Table). In both hill and alluvial fan strata, succulent annuals only accounted for 2% of the plants counted (S10 Table). Only a single succulent plant (*Eremothera boothii*) was registered in the stream channel stratum (S10 Table).

**Table 9:** Estimates of abundance and relative abundance of annual plants during the third phenological period (1 June–30 June) at the Desert Tortoise Research Natural Area, eastern Kern County, California.

**Species**	**Abundance per m^2^**	**Relative abundance (%)**
Schismus barbatus*	124.81	27.76
Lasthenia californica*	78.91	17.55
Pectocarya spp.*	70.20	15.61
Erodium cicutarium*	29.58	6.58
Oxytheca perfoliata*	27.83	6.19
Chorizanthe brevicornu*	17.16	3.82
Amsinckia tessellata*	16.12	3.59
Eriogonum angulosum*	11.25	2.50
Cryptantha circumcissa*	7.17	1.60
Gilia minor*	7.06	1.57
Stylocline psilocarphoides*	4.88	1.08
Eriastrum eremicum*	4.34	0.97
Eriogonum nidularium*	4.01	0.89
Chaenactis carphoclinia*	3.82	0.85
Chorizanthe watsoni*	3.73	0.83
*Eriastrum eremicum*	3.23	0.72
*Eriogonum nidularium*	2.98	0.66
Plantago ovata*	2.84	0.63
Linanthus parryae*	2.66	0.59
Chorizanthe rigida*	2.63	0.58
Caulanthus lasiophyllus*	2.51	0.56
Cryptantha nevadensis*	2.45	0.55
Phacelia tanacetifolia*	2.31	0.51
Mucronea perfoliata*	2.17	0.48
Eriogonum gracillimum*	1.76	0.39
Centrostegia thurberi*	1.60	0.35
Malacothrix coulteri*	1.58	0.35
*Eriogonum angulosum*	1.33	0.30
Loeseliastrum schottii*	1.30	0.29
*Eriogonum gracillimum*	1.18	0.26
Eriophyllum pringlei*	0.92	0.20
Chaenactis fremonti*	0.83	0.18
Mentzelia spp.*	0.78	0.17
Unknown grass*	0.61	0.14
Astragalus didymocarpus*	0.49	0.11
*Eremothera boothii*	0.44	0.10
Nemacladus spp.*	0.43	0.10
Prenanthella exigua*	0.38	0.08
Phacelia fremonti*	0.35	0.08
Linanthus dichotomus*	0.30	0.07
Gilia sp.*	0.29	0.06
Eriogonum pusillum*	0.15	0.03
Syntrichopappus fremonti*	0.06	0.01
*Erodium cicutarium*	0.06	0.01
Eremothera boothii*	0.04	0.01
*Eriogonum pusillum*	0.03	0.01
Total	449.56	100.00

Plant names followed by an asterisk were in a dried state (i.e., had already flowered and fruited—see Materials and Methods).

In the last few weeks of spring, tortoise activity sharply declined, as the majority of our study animals concluded springtime foraging activities by moving into their burrows. During this time, we located only three tortoises (one male and two females) above ground and recorded foraging data for five foraging routes (S11 Table). Two routes consisted of travel segments in one stratum, whereas the remaining three included segments from two strata. The relative distances traveled through each stratum were as follows: 23.5% in alluvial fans, 39.1% in hills, and 37.4% in stream channels (all of which were in small stream channels).

The male tortoise fed primarily on the herbaceous perennial *C. albomarginata* (85% bites), whereas the two females mostly ate dried individuals of the annuals *Eriastrum eremicum* (26% bites) and *Schismus barbatus* (100% bites). Interestingly, the majority of bites taken by one female were from a single dried carcass of an adult Leopard Lizard (*Gambelia wislizenii*; S11 Table).

Together, the three tortoises were observed taking a total of 7,009 bites from 404 individual plants representing at least 15 plant species (11 annuals and 4 perennials; Table 10). In contrast to the first and second phenological periods, tortoise diet included dried plants (78% succulent vs. 22% dried; Table 10). Also in contrast to the first two phenological periods, these tortoises consumed non-plant food items including the dead Leopard Lizard and tortoise scats (Table 10).

**Table 10:** Foods eaten by tortoises during the third phenological period (1 June–30 June) at the Desert Tortoise Research Natural Area, eastern Kern County, California.

**Species**	**# Bites**	**# Plants**	**% Bites**	**% Plants**
***Chamaesyce albomarginata***	3,601	97	51.38	24.01
Eriastrum eremicum*	488	52	6.96	12.87
*Erodium cicutarium*	461	62	6.58	15.35
*Eremothera boothii*	366	35	5.22	8.66
Erodium cicutarium*	258	41	3.68	10.15
*Chorizanthe brevicornu*	257	8	3.67	1.98
Schismus barbatus*	194	26	2.77	6.44
Chorizanthe brevicornu*	119	15	1.70	3.71
Amsinckia tessellata*	116	12	1.66	2.97
Stylocline psilocarphoides*	99	12	1.41	2.97
***Mirabilis laevis***	83	2	1.18	0.50
Cryptantha circumcissa*	34	5	0.49	1.24
Phacelia tanacetifolia *	28	1	0.40	0.25
Oxytheca perfoliata*	25	3	0.36	0.74
*Eriastrum eremicum*	23	5	0.33	1.24
***Astragalus layneae***	7	1	0.10	0.25
Eremothera boothii*	6	3	0.09	0.74
***Ambrosia salsola***	4	1	0.06	0.25
Linanthus parryae*	4	1	0.06	0.25
dead lizard (*Gambelia wislizenii*)	695	-	9.92	-
Unknown plant spp.	105	22	1.50	5.45
tortoise scat	36	-	0.51	-
Total	7,009	404	100.00	100.00

Plant names in boldface are herbaceous perennials; plant names followed by an asterisk were in a dried state (i.e., had already flowered and fruited—see Materials and Methods); # Plants refers to the total number of individual plants for a given species that tortoises fed from (i.e., a plant was counted if a tortoise made at least one bite from that plant). Tortoise scat was eaten on two different occasions by two different tortoises (one male and one female).

**Fig 7:**
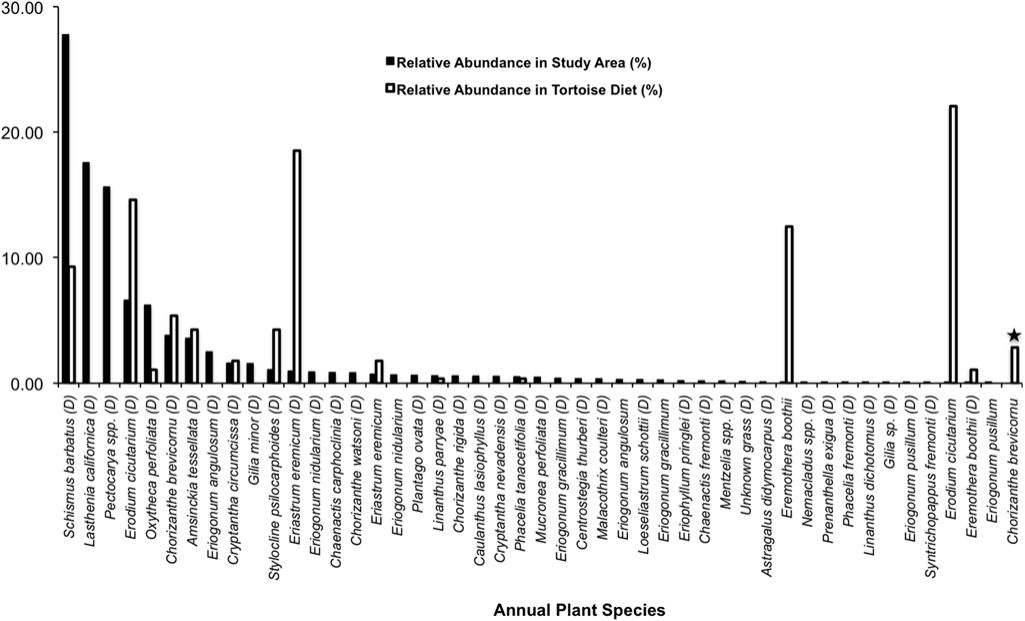
Tortoise diet in relation to annual plant abundance during late-spring. Solid black bars show the relative abundance of each species found in the study area, whereas white bars indicate the relative abundance of each species in tortoise diet during the same period. Star symbols above bars denote plants in tortoise diet that were not detected in the plant survey. (D) indicates that the plants were in a dried phenological state.

**Fig 8:**
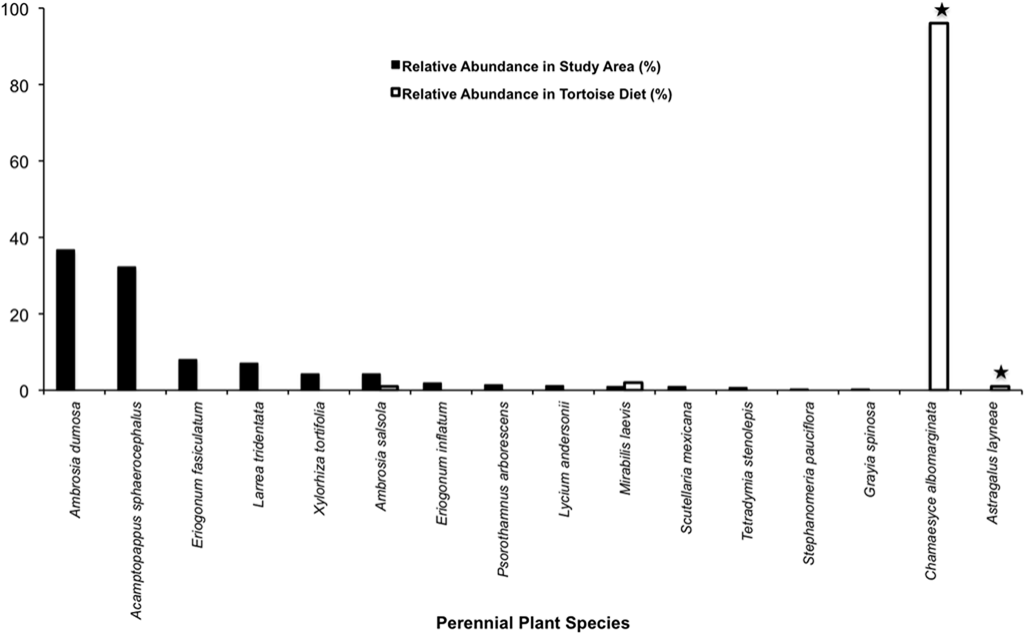
Tortoise diet in relation to perennial plant abundance during late-spring. Solid black bars show the relative abundance of each species found in the study area, whereas white bars indicate the relative abundance of each species in tortoise diet during the same period. Star symbols above bars denote plants in tortoise diet that were not detected in the plant survey.

When comparing plant availability vs. tortoise diet, we see again that tortoises continued to be highly selective foragers of annual and perennial plants (*P* = 0.0005, *P* = 0.0015, respectively; Figs. 7–8; S12–S13 Tables). Our plant sampling efforts documented 39 species of annuals in dried states, seven species of annuals in succulent states, and 14 perennial species; yet we failed to detect several of the tortoise food plants that were still in a succulent state including *C. albomarginata*, *Chorizanthe brevicornu*, and *A. layneae* on the surveys (S12–S13 Tables). Tortoises took bites from four species of annuals in a succulent state and from 11 species of annuals in a dried state.

### Dietary summary of the entire foraging season

**Fig 9:**
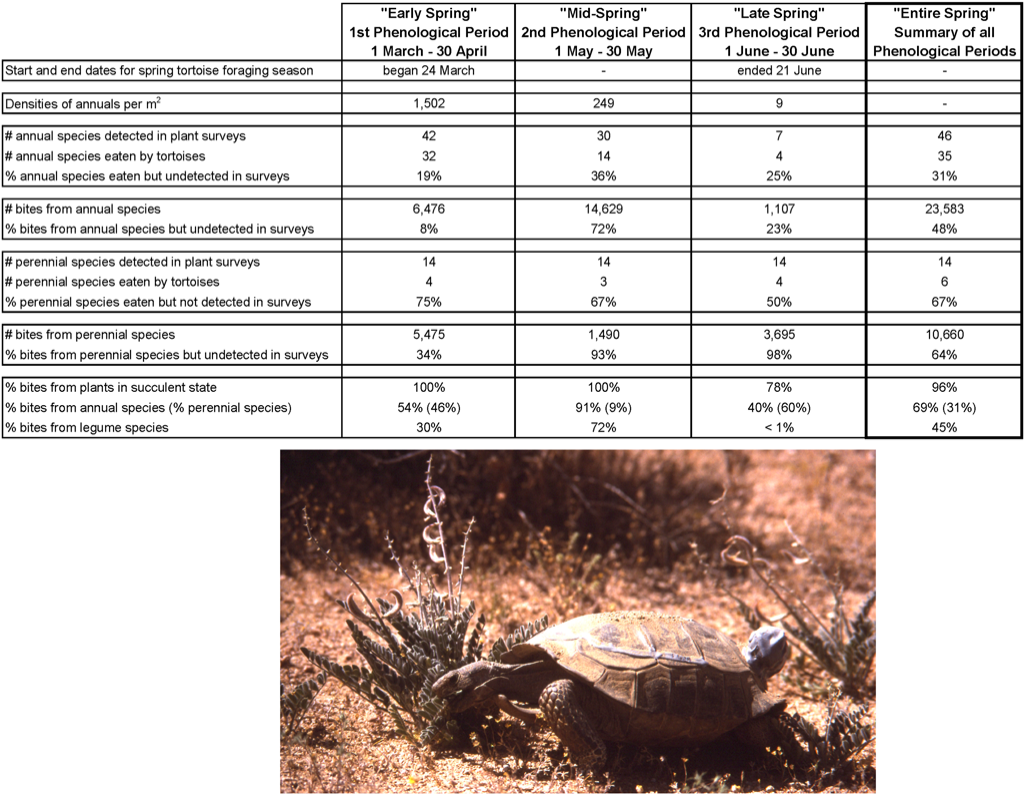
A snapshot of food plant availability and desert tortoise dietary selections throughout the spring foraging season. All summary statistics were derived from Tables 4–10 and plants in the “Unknown plant spp.” category were not included in the estimates. Also, because tortoises largely favored plants in a succulent state, the numbers under the 3rd Phenological Period only represent annual plants that were in a succulent state. The photo shows an adult male desert tortoise eating from an individual *Astragalus layneae*. This plant species, which is a legume and herbaceous perennial, was one of the most preferred food plants during this study. As shown in the photo, these plants often grow in clusters of several individuals. The gray object on the posterior end of the tortoise’s shell is a radio telemetry transmitter (photo credit: W.B. Jennings).

Table 11 provides a detailed accounting of foraging observations for the 18 adult desert tortoises in our study. A total of 35,388 bites were recorded from food items, which included 23,583 bites from annual plants, 10,660 bites from herbaceous perennial plants, 695 bites from a dead lizard, 414 bites from unidentified plants, and 36 bites from tortoise scat. A portrait of the spring foraging season is shown in Fig. 9. Foraging commenced on 24 March and concluded by 21 June with almost daily feeding activities occurring within this time interval. The highest density and species richness of succulent annual plants, as well as total number of different annual species eaten by tortoises, all peaked in early spring and then declined towards the end of spring. If we consider the three phenological periods together as an entire spring foraging season, we see that 46 annual and 14 perennial plant species were found in plant surveys, yet 31% of the annual and 67% of perennial species eaten by tortoises were not detected in the surveys. Moreover, 48% bites from annuals and 64% from perennials were from undetected plant species. Overall, about two-thirds of the bites were from annual species while one-third were from perennials. Plants in a succulent state comprised 96% of bites from plant materials and 45% of bites were from legumes. One of our more striking findings concerns the observation that only two species of legumes, *Acmispon brachycarpus* and *Astragalus layneae*, accounted for more than 73% of the plants eaten and 71% bites taken during the second phenological period.

**Table 11:** Foods eaten by adult desert tortoises during during spring 1992 (24 March–21 June) at the Desert Tortoise Research Natural Area, eastern Kern County, California.

**Species**	**No. Bites**	**% Bites**
*Acmispon brachycarpus*	10,512	29.70
***Mirabilis laevis***	3,820	10.79
***Chamaesyce albomarginata***	3,801	10.74
***Astragalus layneae***	2,902	8.20
*Prenanthella exigua*	1,977	5.59
*Astragalus didymocarpus*	1,623	4.59
*Erodium cicutarium*	1,391	3.93
*Eremothera boothii*	1,367	3.86
*Chorizanthe brevicornu*	919	2.60
*Phacelia tanacetifolia*	710	2.01
*Amsinckia tessellata*	683	1.93
*Mentzelia spp.*	576	1.63
*Cryptantha circumcissa*	561	1.59
*Eriastrum eremicum*	516	1.46
*Plantago ovata*	430	1.22
*Gilia minor*	342	0.97
*Stylocline psilocarphoides*	334	0.94
*Tetrapteron palmeri*	266	0.75
*Schismus barbatus*	244	0.69
*Malacothrix coulteri*	217	0.61
*Lupinus odoratus*	211	0.60
***Stephanomeria parryi***	131	0.37
*Malacothrix glabrata*	125	0.35
*Chaenactis fremontii*	119	0.34
*Pectocarya spp.*	88	0.25
*Loeseliastrum schottii*	79	0.22
*Tropidocarpum gracile*	75	0.21
*Linanthus dichotomus*	66	0.19
*Allium fimbriatum*	25	0.07
*Oxytheca perfoliata*	25	0.07
Unknown grass sp.	18	0.05
*Pholistoma membranaceum*	14	0.04
*Chorizanthe rigida*	12	0.03
*Eriogonum gracillimum*	12	0.03
*Eriogonum pusillum*	12	0.03
*Bromus madritensis*	9	0.03
*Caulanthus inflatus*	8	0.02
*Calycoseris parryi*	7	0.02
*Astragalus acutirostris*	5	0.01
***Ambrosia salsola***	4	0.01
*Linanthus parryae*	4	0.01
***Lomatium mohavense***	2	0.01
*Chaenactis carphoclinia*	1	0.00
dead lizard (*Gambelia wislizeni*)	695	1.96
unidentified plants	414	1.17
tortoise scat	36	0.10
Total	35,388	100.00

Plant names in boldface are herbaceous perennials.

## Discussion

### Desert tortoises in the western Mojave Desert are selective herbivores

The western Mojave Desert received above normal precipitation during the late winter and early spring of 1992 due to an El Niño event, which subsequently resulted in the flowering of at least 78 species of plants known from the DTRNA [24]. This tremendous availability of plants presented the local tortoise population with a broad array of plant species from which to choose their forage. With this rich diversity of potential food items, the adult tortoises apparently foraged 55% (43/78) of annual and perennial species. However, most of the recorded bites (~77%) were on ~10% of the plant species (8/78). Tortoises consumed each of the plant species in different proportions and times during spring depending on their species-specific flowering phenologies. When our dietary data are examined in light of the availability of these plants during different phenological periods of spring, we find strong support for the hypothesis by Jennings [14] that desert tortoises are selective herbivores that track the phenologies of their food plants.

Researchers have identified other species of tortoises as selective foragers on plants, e.g., the giant tortoises, *Geochelone gigantea*, (now *Aldabrachelys gigantea*) of Aldabra atoll [25, 38]; Mediterranean spur-thighed tortoise, *Testudo graeca graeca*, in Morocco [39]; mountain tortoise (*Geochelone pardalis* now *Stigmochelys pardalis*) and serrated tent tortoise, *Psammobates oculifer* [40]. Still other species and populations appear to fall midway between generalist and specialist, e.g., gopher tortoise, *Gopherus polyphemus* [26], leopard tortoise, *S. pardalis* [41], and steppe tortoise, *Testudo horsfieldii* [42]. Tortoises may also alter diets depending on what is available in drought years versus years of abundant forage. Some tortoises may be omnivorous, e.g., Speke’s hinge-back tortoise, *Kinixys spekii* [43].

### Local distributions of preferred food plants

To assess the availability of potential food plants, we stratified our plant sampling at the level of habitat strata and by microhabitat type within strata. Despite placing our sampling quadrats in many, if not all, of the distinctive habitats on the study area, 15 species of plants consumed by tortoises still evaded detection during our plant surveys. Interestingly, this number includes three of the four more frequently eaten species: *Acmispon brachycarpus*, *Chamaesyce albomarginata*, and *Astragalus layneae*. Are these species actually rare in the study area or did inadequate or flawed sampling practices lead to underestimates of their true availabilities? Our casual observations made by frequent walks around the study area confirmed the rarity of these plants. This begs the question: How did tortoises locate these favored plants?

The herbaceous perennial *M. laevis*, the second most-consumed plant species (Table 11), grows only on the rocky portions of the hill stratum. This may help explain why tortoise foraging routes during the first two phenological periods were primarily contained within this stratum (68% and 88% of distances traveled, respectively). In contrast to *A. layneae*, *E. boothii*, and *C. albomarginata*, individuals of *M. laevis* grow singly at each location and are usually separated from other individuals of the same species by tens of meters. However, because *M. laevis* is similar in stature to a small spherically-shaped shrub (~0.3 m tall), it may be visually identifiable from a greater distance than the aforementioned smaller food plants. Also, given the relatively large size of *M. laevis* compared to other preferred food plants, tortoises may only need to find one or several of these plants to satisfy their food intake needs, whereas adult tortoises may need to consume much larger numbers of individuals for smaller-sized annuals. On finding a single *M. laevis*, tortoises typically took hundreds of bites from each plant before moving on, whereas with preferred annual plants and smaller-sized herbaceous perennials, tortoises usually took dozens of bites from each plant.

Tortoises located individuals of *A. layneae* simply by walking along small stream channels (< 2 m wide) until a cluster of this preferred species was encountered. This herbaceous perennial was often found in groups of several plants and is known to grow in clusters of individual plants that are connected underground by their rhizomatous root systems [33]. We confirmed the efficacy of this search strategy of tortoises by selecting random stream channels and walking along them; *A. layneae* clusters can routinely be found in this manner. We observed tortoises to return to the same plants to feed on different days both in 1991 [13] and in 1992 (this study), and we observed one tortoise feed on the same plants *between* years. These observations raise the possibility that tortoises learn the locations of these plants and make repeated trips to feed upon them. Tortoises used a similar strategy to find single individuals or clusters of other preferred plants such as *Eremothera boothii* and *Chamaesyce albomarginata*. Thus, by concentrating their search efforts within particular habitats such as in the hill stratum or in small stream channels, tortoises may increase their chances for locating favored food plants.

Other commonly-selected forage plants tended to grow in patches. These forage plants were locally abundant within patches, with each patch being comprised of dozens or hundreds of individual plants of the target species packed together in areas of ~10 to 100 m^2^. For example, in middle May when the annual *Acmispon brachycarpus* flowered and hence became available as forage, nearly all tortoises focused their foraging efforts on this species, making daily trips to the same patches until these annual plants senesced near the end of May. Only three areas within the study area contained such patches of *A. brachycarpus*, suggesting that the patches themselves may be rare. Also, multiple tortoises were observed to have permanent burrows situated at the edge of these *A. brachycarpus* patches, allowing for easy access to the patches. Daily foraging bouts at this time began with the tortoise leaving its burrow to forage only within the patch then return to the same burrow later in the day. Given that tortoises may use the same burrows across years [44], perhaps these tortoises utilize the same food patches in different years as well. Tortoises similarly utilized patches of the annuals *Astragalus didymocarpus*, *P. exigua*, and *Chorizanthe brevicornu* as well as *Chamaesyce albomarginata* whenever they were encountered.

We considered additional questions regarding rarity of some forage species. Are these plants naturally rare or have they, over time, become rare because tortoises have favored them over other species and consumed them before they could set seed? A precedent exists regarding the latter possibility, as overgrazing by the giant tortoises on Aldabra atoll contributed to depletion of their food supplies and to population declines [45–47]. Might a similar pattern occur with desert tortoises? Although we do not have sufficient data to answer these questions, we note that some of the most frequently eaten plants seem to be habitat specialists, which is a life history trait that can help explain their local rarity. For example, *Astragalus layneae* apparently grows only in and along the margins of small stream channels, which are scattered throughout the study area. As pointed out earlier, an observer walking along one of these channels will inevitably encounter many of these plants, but will rarely find them outside the channels. There is no reason to believe that tortoises selectively extirpated *A. layneae* from non-stream channel habitats first, which collectively cover about 90% of the study area, while leaving individuals of *A. layneae* in stream channels relatively untouched. Similarly, *Mirabilis laevis* and *Prenanthella exigua* show habitat specificity for the hill stratum. However, this argument does not explain the local distribution and abundance of *A. brachycarpus* or *C. albomarginata*, both of which occur in patches that are seemingly located in random locations within the study area. Other possible causes for the local rarity of some preferred forage plants exist. For example, heavy grazing by livestock and other disturbances on the study area prior to the establishment of a protected natural area could have altered the composition and relative abundance of local plants [10, 48]. Future studies should attempt to disentangle these various hypotheses, which will hopefully better determine the causes of food plant rarity in this area.

The quality of an inference about diet selection can be adversely affected by how an investigator defines “availability” with respect to the diet of the focal animal species [19]. In this study, we defined availability as all plants occuring across the study area, an area encompassing home ranges of our study animals, not including habitats devoid of tortoises. Our definition of availability seems to have been reasonably correct given that our study animals often traveled among habitat strata while following their daily foraging routes. Had we instead assessed availability along the foraging routes of desert tortoises, as has been the practice in previous desert tortoise foraging studies [13, 18], it is possible that these rare plants—especially those that grow in patches—would instead appear more frequently in the plant surveys and thus give rise to a misleading impression about their availabilities to tortoises. In other words, the higher order selection by the tortoises (choosing the patch in the first place) would be ignored. Such estimates of plant availability determined from foraging paths could lead to the wrong conclusion that tortoises are not selecting particular species of plants. This illustrates the problem in which investigators could obtain spurious results if they viewed selection primarily at one level (i.e., within a feeding site or 3^rd^ order selection) when in reality the animals are first selecting at a higher level (i.e., home range or feeding sites)[19]. In the present study, our findings suggest that desert tortoises are exhibiting a combination of 2^nd^ (i.e., selecting strata and feeding sites at patches of favored plants) and 3^rd^ order selection (i.e., selecting particular plant species within strata or feeding sites consisting of patches of favored foods).

### Significance of herbaceous perennials in desert tortoise diet?

In spite of the overwhelming abundance of winter-spring annuals that flowered throughout spring, several species of herbaceous perennials nonetheless composed ~30% of tortoise diet. Aside from any nutritional benefits that may be accrued, herbaceous perennials may play an important role in maintaining tortoise health, particularly during drought years. For example, the species known to be tortoise forage plants at the DTRNA and elsewhere where desert tortoises occur (i.e., *Astragalus layneae*, *A. lentiginosus*, *Chamaesyce albomarginata*), have the ability to regenerate their stems, leaves, and flowers following rainfall outside of the normal winter and spring flowering seasons (WBJ and KHB pers. observations). In contrast, most native winter-spring annuals cannot germinate—hence become available as forage for tortoises—if winter rainfall is insufficient or arrives too late to stimulate germination and flowering [23]. Thus, if the winter-spring annuals fail to germinate during drought years, then rainfall events occurring in late spring or during summer can initiate re-growth of herbaceous perennials, thus providing tortoises with much needed food and water. Evidence in support of this hypothesis comes from the study by Beatley [49] who found that an herbaceous perennial (*Astragalus lentiginosus*) sustained kangaroo rat (*Dipodomys merriami*) populations in southern Nevada during drought years.

### Why are desert tortoises selective foragers?

Scientists have discussed optimal foraging theory in different trophic groups of animals for decades; the discourse on large herbivores (ungulates) is particularly relevant and can be applied to the tortoise, a small herbivore [50]. The fact that a number of adult tortoises independently selected the same locally-rare plant species to be their primary food choices from amongst the vast number of available plant species shows how selective desert tortoises can be. This behavior implies that these particular species offer significant nutritional benefits to desert tortoises. Why are tortoises targeting these plant species? What are the nutritional benefits of these selected plants and how do they differ from other available plants?

Interestingly, three of the preferred plants *Acmispon brachycarpus*, *Astragalus layneae*, and *A. didymocarpus* were leguminous plants, a finding that mirrors the results of an earlier study at the DTRNA [13]. These three species alone comprised more than 42% of bites taken from all foods during the spring foraging season (Table 11). This result may not be that surprising given that leguminous plants appear to be important constituents in desert tortoise diet over much of their range within the Mojave Desert (KHB, unpublished data). Other tortoise species also consume legumes [26, 30, 39, 40, 41, 51] but see [42]. This conspicuous consumption of legumes by tortoises is noteworthy because legumes are well known to contain high levels of nitrogen in their tissues owing to their symbiotic relationship with nitrogen-fixing bacteria in the soil [52].

Although plants containing high levels of nitrogen are expected to be beneficial to tortoise growth and health, Oftedal [17] and Oftedal et al. [18] suggest that nutritiousness of tortoise food plants should be primarily evaluated on the basis of their combined nitrogen, water, and potassium content. This is because tortoises usually face deficits of not only nitrogen but also water and perhaps other nutrients [18]. Moreover, desert plants often contain high levels of potassium, which in high concentrations can be toxic to tortoises [17, 53]. Unlike other desert-dwelling herbivorous reptiles such as Desert Iguanas (*Dipsosaurus dorsalis*) and Chuckwallas (*Sauromalus obesus*), which can excrete excess potassium via salt glands, desert tortoises do not have salt glands and therefore can only excrete excess potassium via urine production or urate precipitates [17, 18, 54]. However, eliminating excess potassium via urination wastes water, a nutrient not available to desert tortoises for much of the year or even over multi-year droughts [6, 17, 18]. Likewise, the production of urates to remove excess potassium can have a strong negative impact on the nitrogen budgets of tortoises, particularly if potassium concentrations in tortoises reach sufficiently high levels relative to water and protein concentrations [17, 18].

Given the physiological interactions among water, protein, and potassium, Oftedal et al. [18] predicted that free-ranging desert tortoises should choose plant foods that have high water and protein concentrations and low potassium concentrations. They found that the foods consumed by juvenile tortoises were higher in protein (but not higher in water) and lower in potassium than the plants they bypassed along their foraging routes, thereby providing evidence supporting their hypothesis. The negative impacts to nutrition by tortoises eating poor-quality food plants were first illuminated in the studies by Meienberger et al. [55] and Peterson [56], each of which documented weight losses and negative nitrogen balances by adult desert tortoises following the consumption of dried grasses in summer. A more recent experimental study showed that juvenile desert tortoises also lost mass as a result of eating dried grasses, yet juveniles gained mass when eating succulent forbs [57]. These studies highlight the importance of nitrogen to both juvenile and adult desert tortoises and show that this nutrient could only be acquired from succulent green forbs and not from dried grasses.

Although nitrogen, water, and potassium are important components to desert tortoise nutrition, other nutrients, particularly calcium and phosphorus, may also be limiting to tortoises [17, 58]. Hazard et al. [58] observed decreases in shell volume of juveniles, which they attributed in part to the loss of phosphorus by eating dried grasses. Hazard et al. [58] further hypothesized that the energy gained by juveniles from eating dried summer grasses may be outweighed by the negative consequences of losing phosphorus and nitrogen. Moreover, these authors suggested that deficiencies of calcium and phosphorus have two important consequences to desert tortoise nutrition and ecology. First, deficiencies could lead to insufficient mineralization of the shells in juveniles, which could increase their vulnerability to predation and, secondly, could limit egg production by adult females.

Developing a comprehensive understanding of the nutritional value of annual plants to desert tortoises will be challenging for a number of reasons. First, tortoise habitats contain a large number of annual species, which presents a significant barrier to obtaining a complete catalog of their nutrient compositions. In the present study, the total number of ephemeral species either sampled by us or by the tortoises exceeded 60 species. Many other species of ephemerals exist in tortoise habitat elsewhere in the Mojave Desert. Secondly, the nutrient composition of an ephemeral plant can vary at different levels. Plants of the same species can vary across populations if the nutrient composition of soils varies among geographic regions. Nutrient composition may also depend on the phenological state of the plant [17, 58] and on which plant structure is assayed (e.g., stems, flowers, seeds; [17, 57, 58]).

Once the nutrient composition of a plant is determined, challenges remain because relating the estimates for the various component nutrients (e.g., nitrogen, phosphorus) and physiologically relevant plant structures (e.g., fiber content) to tortoise nutritional requirements can be difficult. For example, the benefits of a large amount of a “good” nutrient such as nitrogen can be offset by the presence of a large amount of a “bad” nutrient such as potassium [17] or a high content of fiber can decrease the effective availability of a key nutrient even if the plant contains a high amount of this nutrient [57]. Despite these complications, these early nutritional studies are promising and should be expanded to include both juvenile and adult tortoises throughout their geographic range and cover the entire foraging season in both wet and dry years.

### Rarity of tortoise food plants and conservation implications

In view of the status of Agassiz’s desert tortoise as a threatened species, the findings of this study have important conservation implications for tortoises living in the western Mojave Desert, other parts of its geographic range, and for other closely-related species. If tortoises have evolved a dependence on particular forage species, then any factor that causes decline or extirpation of these apparently limited food resources could also adversely affect the tortoises. One factor that likely negatively affects the local abundance of the native annual plants is competition from introduced plant species [59]. At the DTRNA, which was fenced and protected starting in 1980, Brooks [60] showed that alien plant abundance was lower within the preserve compared to areas outside. Still, even in the protected confines of the DTRNA, alien plants are dominant, as only two species—*Schismus barbatus* and *Erodium cicutarium*—represented about 30% of the annual flora during the first phenological period in this study. In a more extensive study of the annual flora outside of the DTRNA, but within designated Critical Habitat in the western Mojave Desert, Brooks and Berry [10] found that alien plants comprised 6% of the flora and 66% of the biomass during a high rainfall year, whereas in a dry year these numbers were elevated to 27% and 91%, respectively. It is well known that exotic species thrive in disturbed habitats [7]. Thus agents of disturbance such as livestock grazing [48, 61], off-road vehicle use [62, 63], or fire [15, 64] may contribute to the proliferation of exotics to the point of competitively excluding species of native plants critical to tortoise diet. These agents of habitat degradation can also act to directly destroy tortoise food plants.

Climate change is another factor that could negatively affect the distribution and abundance of critical tortoise food plants [65] and, in turn, impact the well being of tortoise populations. In support of this idea, a recent paleo-survey of the arctic tundra flora and diets of its dependent megafauna suggest that climate-induced change from a forb-dominated flora to a grass-dominated flora may have caused or hastened the extinctions of such animals as the Woolly Mammoth (*Mammuthus primigenius*) [66]. In summary, the U.S. Fish and Wildlife Service [4], in the first recovery plan for Agassiz’s desert tortoise, recommended that land managers and faunal conservation authorities prohibit activities that negatively affect the integrity of tortoise habitat, e.g., livestock grazing, off-road vehicle activity, and other habitat destructive activities.

## Conclusions

Our findings provide the strongest evidence to date supporting the hypothesis that desert tortoises in the western Mojave Desert are selective herbivores. Although most of the more commonly eaten food plants were locally rare, tortoises employed effective search strategies to locate these food resources. Desert tortoises in this region focused much of their foraging efforts on several locally-rare leguminous species, which may play an important role in tortoise health especially in their growth [17, 18]. Another novel finding of this study is that much of tortoise diet was comprised of herbaceous perennials, which in contrast to the more abundant winter-spring annuals, may help sustain tortoise populations during drought years. Any factor that directly or indirectly reduces the availability of key tortoise food plants (e.g., offroad vehicle use, livestock grazing, or climate change) may negatively affect tortoise populations.

## Supporting Information

S1 Table Perennial density and cover (data, calculations).v3. (XLSX)

S2 Table Annual abundance first phenological period (data, calculations).v2. (XLSX)

S3 Table Diet data for individual tortoises first phenological period.v2. (XLSX)

S4 Table Fisher’s Test annuals vs. diet first phenological period.v3. (XLSX)

S5 Table Fisher’s Test perennials vs. diet first phenological period.v3. (XLSX)

S6 Table Annual abundance second phenological period (data, calculations).v3. (XLSX)

S7 Table Diet data for individual tortoises second phenological period.v3. (XLSX)

S8 Table Fisher’s Test annuals vs. diet second phenological period.v3. (XLSX)

S9 Table Fisher’s Test perennials vs. diet second phenological period.v3. (XLSX)

S10 Table Annual abundance third phenological period (data, calculations).v2. (XLSX)

S11 Table Diet data for individual tortoises third phenological period.v3. (XLSX)

S12 Table Fisher’s Test annuals vs. diet third phenological period.v3. (XLSX)

S13 Table Fisher’s Test perennials vs. diet third phenological period.v3. (XLSX)
